# Self-Incompatibility in Devil’s Potato (*Echites umbellatus* Jacq., Apocynaceae) May Explain Why Few Flowers Set Fruit

**DOI:** 10.3390/biology13060423

**Published:** 2024-06-07

**Authors:** Suzanne Koptur, Andrea Salas Primoli, Imeña Valdes, Maha Nusrat

**Affiliations:** 1 Department of Biological Sciences, International Center for Tropical Botany, Institute of the Environment, Florida International University, Miami, FL 33199, USA; asala035@fiu.edu (A.S.P.); imenavaldes2020@u.northwestern.edu (I.V.); mahanusrat@gmail.com (M.N.); 2 Program in Plant Biology and Conservation, Northwestern University, 2145 Sheridan Road, Tech F315633, Evanston, IL 60208, USA

**Keywords:** breeding system, Caribbean, compatibility, Florida, flowers, fruits, hawkmoths, pine rocklands, pollination, seeds

## Abstract

**Simple Summary:**

*Echites umbellatus* is a plant with tubular white flowers that are pollinated by hawkmoths. Few fruits are produced in nature, so we investigated the breeding system of *E. umbellatus* by growing plants from different populations in a greenhouse and hand-pollinating flowers with self-pollen, pollen from siblings (plants grown from seeds in the same fruit), and pollen from plants in other populations. Pollinations between unrelated plants were most successful, and we conclude that this species is mostly self-incompatible, though most populations had a few individuals that were somewhat self-compatible. There were more self-compatible individuals in smaller habitat fragments, perhaps a function of limited mating opportunities. We conclude that self-incompatibility in this species contributes to its limited fruit set, though other factors such as low pollinator activity may also be important.

**Abstract:**

Pollinators are needed for the reproduction of *Echites umbellatus*, and only sphingid moths have mouthparts long enough to reach the nectar at the bottom of the species’ long, twisted floral tube. Though plants produce many flowers over a period of several months, one observes very few fruits in nature. We asked: (1) Are plants self-compatible, or do they need pollen from another individual to set fruit and seed? (2) Are cross-pollinations between unrelated individuals more successful than crosses with relatives? (3) How does the relatedness of pollen and ovule parent plants affect fruit set, seed number, and seed quality? We investigated the breeding system of *E. umbellatus* by collecting fruits from seven sites, growing plants and performing hand pollinations over a period of several years, collecting and measuring fruits and counting seeds. *Echites umbellatus* is self-incompatible, though some individuals produce fruit by self-pollination. Cross-pollinations between unrelated individuals set the most fruit (59%), and those that were self-pollinated set the least (9%). Fruit set from cross-pollinations between related individuals was intermediate (32%). Although the number of seeds per fruit did not differ significantly among pollination treatments, fruits from self-pollinations had substantially fewer viable seeds than outcrossed fruits, with fruits from sibling crosses being intermediate. There were higher levels of self-compatibility in the fragment populations compared with plants from intact habitats. Self-incompatibility may explain why fruit set is low in this plant species; future investigation into the breakdown of self-incompatibility in smaller populations is warranted.

## 1. Introduction

Most flowering plants need pollinators to accomplish their own sexual reproduction. Flowers have evolved over time to attract and reward visitors, with increasing specialization limiting visitation to the most effective pollinators, as can be seen in many plants pollinated by hawkmoths (Lepidoptera: Sphingidae) [1,2]. Many studies have been undertaken to address the lack of knowledge of the interactions between flowering plants and their invertebrate pollinators [3] and in most parts of the world, pollinator/plant mutualisms are endangered [4]. Declines in insect populations have been the subject of much recent concern and are attributed to a multitude of factors, all contributing to a general decline in environmental quality [5]. Loss of pollinators may have large impacts on biodiversity conservation as well as food production [6,7]. On a more local and regional scale, habitat fragmentation can have measurable effects on insect diversity and pollinators [8] though such changes may display a delayed effect [9] and are not found in every situation [10].

Like many members of the Apocynaceae, *Echites umbellatus* plants produce many flowers but few fruit. This phenomenon suggests that the plants are pollen-limited in some way [11]: either flowers lack pollinators, or the plants receive pollen that is not compatible. If plants are self-incompatible, even if pollen from the plant itself or closely related individuals is deposited, it is less likely to produce fruit than crosses between unrelated individuals. To explore what aspects of compatibility might be related to this low fruit set, we undertook a greenhouse experiment to determine the breeding system of *E. umbellatus* to better understand the reproduction of this widespread and resilient Caribbean species. By raising plants from fruits collected from different populations and keeping track of siblings (plants from seeds of the same fruit which share the same maternal parent), it was possible to compare the results of pollinations within individuals, between related individuals, and between entirely unrelated individuals.

## 2. Materials and Methods

### 2.1. Study Organism

*Echites umbellatus* Jacq. (Apocynaceae) (syn. *Echites umbellata* Jacquin 1760) is a woody perennial vine that is widespread in southeastern Florida and the Caribbean and is found in pine rocklands and coastal habitats. It is one of the first plants to resprout after a fire in the fire-successional pine rocklands habitat and serves as hostplant for some colorful diurnal moths: the oleander moth (or polka dot wasp moth; *Syntomeida epilais*) and the Uncle Sam moth (or faithful beauty; *Composia fidelissima*). It may also host, though not commonly in south Florida [12], the drab-colored tetrio sphinx moth (*Pseudosphinx tetrio*) that has colorful caterpillars said to mimic coral snakes [13].

Various methods of pollen aggregation occur in the Apocynaceae, perhaps in response to a drying climate over evolutionary time [14]. Some have pollinia, like orchids (e.g., *Asclepias* spp.), whereas others have different ways of ensuring that many pollen grains are picked up in one floral visit. In this way, one visit from a suitable pollinator can export enough pollen for fruit to be set when deposited on the stigmatic surface of a compatible conspecific plant, providing great economy in pollination and increasing pollen transfer efficiency [15]. As other members of the large APSA clade (made up of the subfamily Apocynoideae and exemplars of subfamilies Periplocoideae, Secamonoideae, and Asclepiadoideae of the family Apocynaceae [16]), *Echites umbellatus* flowers have a gynostegium, a structure composed of the female style head and the male anthers, facilitating pollen pickup and deposit by floral visitors [17]. Within the floral tube of *E. umbellatus*, spiraling rows of hairs serve to guide the visitor’s proboscis to the nectar at the bottom, and in passing the gynostegium after insertion, the tongue is coated with glue from the sticky sides of the style head. When the tongue is withdrawn, if it has pollen on it, pollen is deposited on the style cuff at the base of the gynostegium, and the tongue picks up pollen that adheres to the recently deposited glue from the flower being visited. The large, white corollas with long floral tubes, along with a fragrance detected only late at night, suggest the flowers of *E. umbellatus* are visited by hawkmoths [18]. By measuring the widths of floral visitor mouthparts and utilizing monofilament fishing line of different widths corresponding to different groups of visitors, we concluded that the best fit for *Echites* flowers are hawkmoth proboscides [19].

In previous fieldwork, we and collaborators have watched flowers for visitors in several locations for many hours [18]. We have never seen a living moth visit the flowers, though our earlier simulation experiments showed that hawkmoths are the only visitors with mouthparts the right size to reach the nectar, remove pollen, and deposit pollen in another flower [19]. Individual flowers of *Echites umbellatus* plants last from 7 to 10 days, giving a long window for visits by hawkmoth pollinators, which may happen infrequently and late at night.

### 2.2. Experimental Procedure

We grew plants from seven populations: two pine rockland fragments (Larry and Penny Thompson Park (L&P); and Navy Wells), four from Everglades National Park (all in Miami-Dade County, Florida, USA; ENP, ENP A, ENP B, ENP C), and one from the San Salvador Island, Bahamas (Bahamas). Keeping track of seeds from the same fruit, we grew sibling cohorts germinating seeds (starting in 2013) individually in divided seed-starting plastic trays, using Pro-mix premium potting mix. From each population, we chose 15 plants that were transplanted and grown to adult size in 3-gallon pots, using three bamboo sticks joined at their top ends to form a framework for each individual plant, around which we twined its vining stems. When the plants began flowering (taking from 14 months to two years in our greenhouse), we performed pollinations over four years (2014–2018), as plants came into flower. Normally, a plant produced one or two flowers per day for several months, though sometimes plants had more, or none. Our goal was to perform five pollinations of each type (self, sibling cross, and outcross) on each of 115 plants, but for many individuals, this number was not reached. If we had more than one flower of each pollination type per plant, it was included in our analysis. Data from plants with no, or too few, pollinations were not included.

To perform hand pollinations, using standard hand-pollination methods [20] (p. 221), we used monofilament fishing lines of the appropriate diameter to remove and deposit pollen [19]. Inserting the line into the opening of the corolla tube, it passed down a spiraling channel to slide by the sticky area on the style head where “glue” was applied; we then withdrew the line that now had its end covered in glue and picked up pollen as the line was drawn up through the pollen chamber formed by the anthers around the top of the style head. This pollen-bearing line was then inserted into the recipient flower. To perform self-pollinations, we inserted a line three times in the same flower—the first time, to get glue on the line and pick up pollen; the second and third times, to deposit pollen on the receptive stigmatic area, below the sticky area, at the base of the style head. Pollinations between plants were performed by using a piece of line, inserting it into a fresh flower on the pollen parent, and then inserting it a single time into the recipient (ovule) parent. Sibling crosses were made between parent plants grown from the same fruit. Crosses were true outcross pollinations, performed between individuals from different populations, (presumably) entirely unrelated. As plants were kept in a pollinator-free greenhouse, we did not cage or bag flowers to prevent visitation, nor did we include an emasculated flower treatment as the floral mechanism does not allow automatic self-pollination.

**Figure 1:**
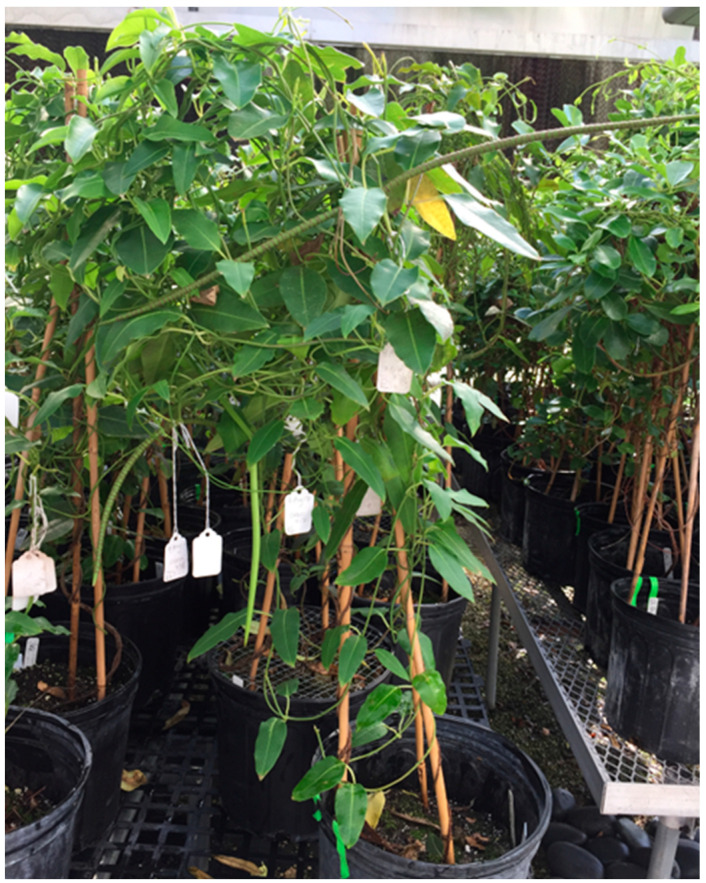
*Echites umbellatus* plants in a greenhouse at FIU. The long follicles of the two-parted fruits are wrapped with thread to prevent seed dispersal prior to collection.

Each pollinated flower was marked with a hanging paper tag labeled with the date, pollination treatment, and pollen parent and was checked off in our task chart. The fate of every pollination was recorded, and when a fruit grew to its maximum size but was still green in color (a process that took 3–5 months), we used strong thread to wind around it to prevent the loss of wind-dispersed seeds upon dehiscence (Figure 1). Individual mature fruits (that had turned dark brown) were collected and gently dried in a drying oven at 30 degrees Celsius in brown paper bags. We measured the length of each of the two follicles on dried fruits. We sorted seeds from each fruit, first removing the fluffy appendages that aid in wind dispersal, and then separating normal-sized seeds (which were filled and plumper) from smaller, unfilled ones that we presumed to be non-viable, recording the numbers of each type.

We performed germination tests to compare seeds resulting from each pollination treatment (18 samples of each type of seed, filled and unfilled, from each pollination treatment on different individual plants), as well as the viability of unfilled seeds. Seeds of *E. umbellatus* germinate well without any pre-treatment [21] and are both dessication- and freezing-tolerant [22], so we simply stored them in seed envelopes after counting them from the dried fruits. Each sample of 20 seeds was started in a covered Petri dish lined with a filter paper disk (Whatman No.1) and distilled water and was placed over a grid to provide equal space to facilitate censusing and discourage mold growth. We recorded germination daily for 21–28 days until maximum germination was reached. We had 18 samples from different individual plants of each type of seed, filled and unfilled, from each pollination treatment.

### 2.3. Data Analysis

We compared the overall number of fruits from different pollination treatments using a contingency table analysis. We calculated the index of self-incompatibility (ISI), a quantitative estimate of the frequency of fruit set with self-pollination compared with that of cross-pollination for individual plants, a method that has been widely used. We used the sum of both sibling and outcross fruit sets from our experiment for cross-pollinated fruit set in our calculations. This index is interpreted in an intuitively opposite way of what its name implies, with higher values of ISI indicating greater self-compatibility. For this reason, others have used the modification of Lloyd [23] and Raduski et al. [24], the inverse of the index, so that higher values of ISI indicate greater self-incompatibility, which is found by calculating ISI as 1 minus the proportion of self-pollinated fruit set/crossed fruit set. We explored the relationship between follicle length and the number of seeds (total, filled, and unfilled) using correlation analysis. Results from the germination experiment were compared among good (filled) seeds using one-way ANOVA with a post hoc Student–Newman–Keuls test with equal variance not assumed.

## 3. Results

**Figure 2:**
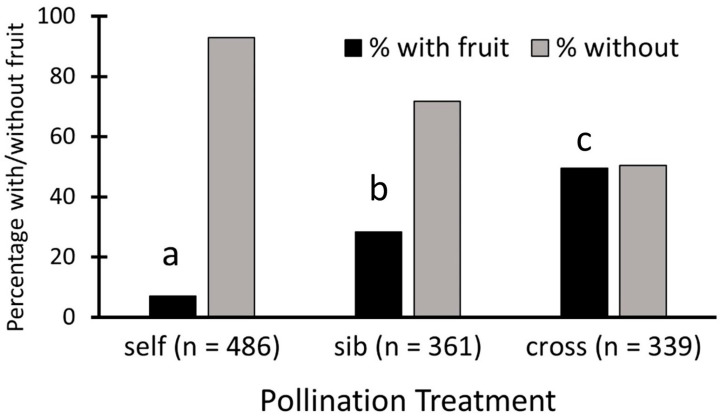
Fruit set for *Echites umbellatus* flowers with different pollination treatments over all populations combined. Each letter above a black bar denotes a treatment with fruit set that is significantly different from those with other letters.

Nearly half of all cross-pollinations, 49.6%, set fruit, 28.3% of sibling crosses set fruit, and only 7% of self-pollinations set fruit (Figure 2). These proportions are significantly different, as shown by the contingency table analysis (Pearson χ^2^_2_ = 191.7, *p* < 0.0001), and each differs from the others (*p* < 0.05).

When sites are considered separately, the patterns are not uniform. Two sites followed the same pattern as the overall one described above (fruit set of self- less than that of sib- and sib- less than outcross: ENP A, Bahamas); others showed no difference between fruit sets with sibling and outcross treatments, but both were more than selfed (L&P, ENP, ENP B, ENP C) and for the remaining site, there was no difference between fruit sets from self and sibling pollinations, but both were less than outcross (Navy Wells). The ENP site was the only one where no fruit at all resulted from self-pollinations, and there was no difference between sibling and outcross fruit sets.

**Figure 3:**
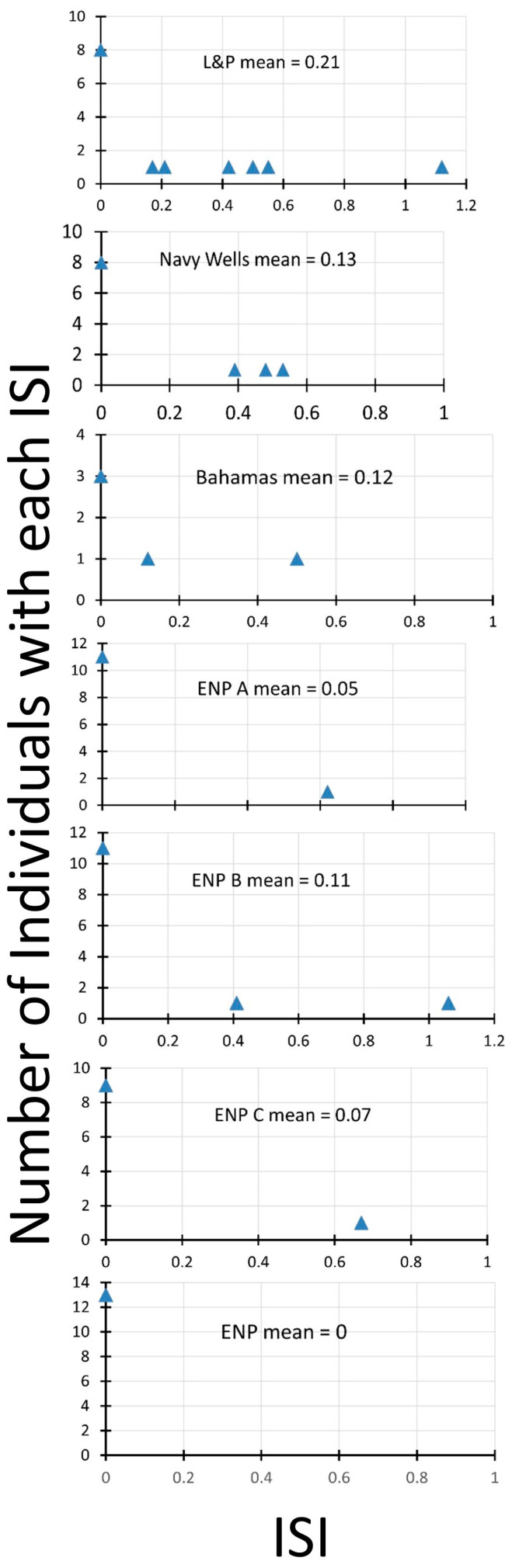
Index of self-incompatibility for seven populations of *Echites umbellatus*, six in south Florida, one in the Bahamas. Everglades National Park sites (ENP, ENP A, ENP B, and ENP C) are in more continuous natural pine rockland habitat in a mosaic of hardwood hammock and finger glade. Larry and Penny Thompson Park (L&P) and Navy Wells are fragments of pine rockland isolated from other natural areas. The Bahamas site (Bahamas) is a coastal site called Sandy Hook on San Salvador Island.

Rather than plot charts of fruit sets from pollination treatments from each site, we present the results in terms of the index of self-incompatibility (ISI). The lower the value of this index, the more self-incompatible an individual plant is. The plot of the distribution of ISIs and the mean ISI in each population (Figure 3) shows differences among the populations. The two south Florida fragment sites have the highest average ISI, with many individuals showing some self-compatibility. The Bahamas site is next, with a large proportion of the few individuals compared showing self-compatibility. One of the Everglades sites (ENP B) has two individuals with self-compatibility, whereas the others have either only one (ENP A, ENP C) or none (ENP).

**Figure 4:**
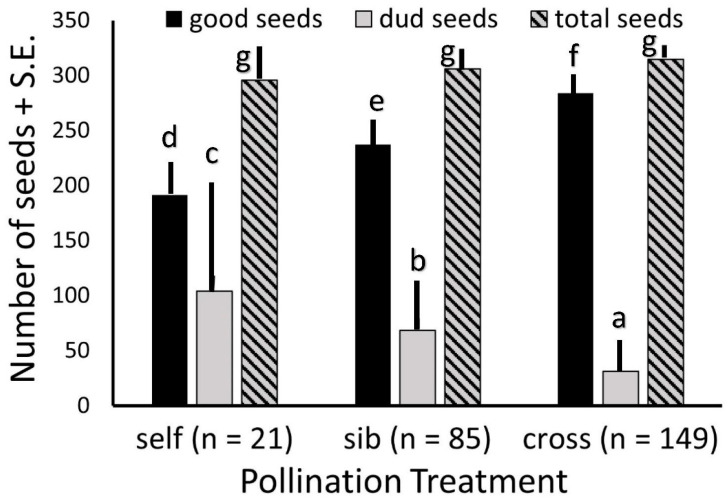
Seed set per fruit of *Echites umbellatus* from different pollination treatments over all populations totals. Each letter above a bar of one category (filled or “good”, unfilled or “dud”, and total) denotes a treatment with seed numbers that is significantly different from those with other letters over the same category bar.

As far fewer fruits were produced from hand self-pollinations, and only half as many from sibling as from outcross pollinations, we considered all populations together when comparing fruit sizes, numbers of seeds, and numbers of viable versus unfilled seeds. The average number of seeds per fruit did not differ among the three pollination treatments. However, fruits from outcross pollinations set substantially more viable seeds than did fruits from sibling pollinations, and both substantially more than did fruits from self-pollinations. Fruits from self-pollinations had substantially more unfilled seeds than did those from sibling pollinations, and both had considerably more unfilled seeds than did outcross fruits (Figure 4).

**Figure 5:**
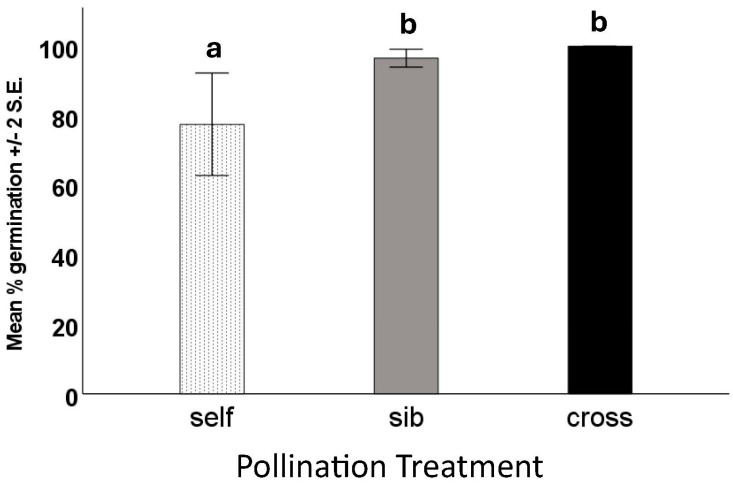
Germination test results of filled seeds from fruits resulting from different hand-pollination treatments of *Echites umbellatus*. Error bars are ±2 S.E. Means with different letters above the bars were shown to be significantly different from one another by ANOVA.

We performed germination tests on viable and unfilled seeds from all pollination types—18 trials, each of twenty of each seed type. As anticipated, none of the “dud” (unfilled) seeds germinated or produced seedlings, whereas nearly all the viable (filled) seeds did. There was no significant difference in either the number of days to first germination or the number of days to maximum germination (F = 7.8, df = 2, *p* = 0.496). The percentage of seeds to germinate did differ among treatments, with seeds from fruits produced by self-pollination showing lower germination than those from fruits produced by sibling or outcross pollination (Figure 5).

**Figure 6:**
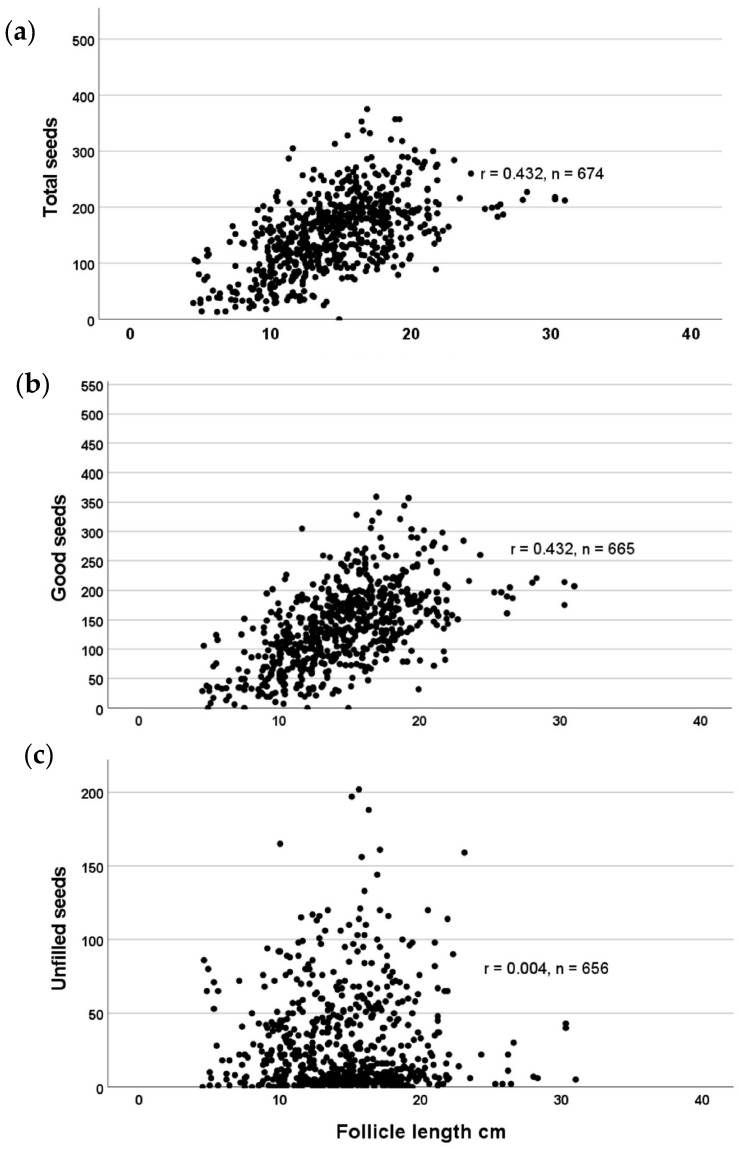
Correlations of *Echites umbellatus* fruit follicle length with (**a**) number of seeds total; (**b**) number of filled seeds; and (**c**) number of unfilled seeds.

There is a significant relationship between follicle length and the total number of seeds contained (Figure 6), as well as the number of filled (viable) seeds (r = 0.432 in both cases). There is no relationship between follicle length and the number of unfilled seeds (r = 0.004).

## 4. Discussion

Outcrossing is normally desirable for flowering plants, as it creates increased vigor [25] and genetic variability that enables plant species to adapt to a changing environment. Flowering plants have a variety of mechanisms to prevent or reduce self-pollination, such as heteromorphy [26], dicliny, and dioecy, as well as self-incompatibility systems [27]. Many species with perfect flowers receive self-pollen either via cleistogamy, automatic selfing, or recurring visits from certain animals; they may also have floral mechanisms such as delayed self-pollination as the corolla falls off [28] or the flower shrivels [29], providing fertilization in self-compatible species. *Echites umbellatus* flowers that were unmanipulated (not hand pollinated) set no fruit, indicating that this species relies on pollinators to reproduce. Similar results have been shown in other Apocynaceae: most species set no fruit when pollinators are excluded from their flowers (*Angadenia berteroi* [30]; *Apocynum cannabinum* [31]; *Aspidosperma quebracho-blanco* [32]; *Mandevilla pentlandiana* [33]; *Mandevilla tenuifolia* [34]; *Nerium oleander* [35]). The low level of fruit set (few fruits resulting from the many flowers produced) by an *Echites umbellatus* plant in the field is likely attributable to self-incompatibility, as most plants in our experiment set no fruit with self-pollination. In all populations, save one, there was at least one individual that showed some self-compatibility, and this number was greater in the fragment populations and the Bahamas than in the Everglades sites. A plant may be physically and physiologically limited as to the number of fruits it sets as well, producing more flowers to attract visitors. Herrera [35] concluded that 80% of flowers of *Nerium oleander* were just for show to attract pollinators, as extensive hand pollination only yielded a maximum of four fruits per inflorescence. Considering all *Echites* populations in our experiment, outcross pollinations (with pollen from another population) produced fruit only half of the time. This may be due to our hand-pollination technique, or perhaps some sort of outbreeding depression [36,37,38]. Self-incompatible Apocynaceae species usually display a low natural fruit set, even with ample pollinator activity [31,39,40,41]. In *Echites umbellatus*, as in *Angadenia berteroi* [30], the highest fruit set resulted from crosses between unrelated individuals. In other Apocynaceae with self-compatibility, in most or only some individuals, fruit set is highest when flowers are cross-pollinated [39,42,43].

Incompatibility may be expressed in many ways, including fruit set, seed set, seed filling, and seed viability. Pollinations performed on *Echites* flowers with pollen from siblings (from seeds produced from the same fruit) were less successful in setting fruit, and although there were the same number of seeds in the fruit produced, fewer of them were viable than in fruits from cross-pollinations. Fruits produced from self-pollinations also had the same number of seeds, but many more were unfilled and inviable. In addition, a lower proportion of the apparently viable seeds germinated from those selfed fruits. Similar to our findings, *Asclepias incarnata* fruits from self-pollinations had a lower total seed mass and a higher proportion of unfilled seeds than fruits from cross-pollinations [44]. *Wrightia tomentosa* also produced more fruits with cross-pollinations than with self-pollinations, and the number of seeds was the same but with a greater seed weight in fruits from cross-pollinations [43], suggesting that more of those seeds were filled and viable.

We found that levels of self-incompatibility varied among the populations of *Echites umbellatus* sampled, with self-fertile individuals ranging from 0% to 43% of the individuals studied. Other studies that have considered multiple populations of Apocynaceae have found similar results: in *Asclepias exaltata*, the proportion of self-fertile individuals ranged from 0 to 34.0% in six populations, differing significantly among populations [31]. Many plants have been shown to differ in outcrossing rates among populations of different species in the same genus [45] as well as among populations of the same species [46].

Greater amounts of self-compatibility in smaller populations (as we found in the fragmented pine rockland sites, with 27–43% of individuals exhibiting self-compatibility, versus 0–15% in Everglades populations) suggest that selfing may be selected when there are fewer opportunities for outbreeding. Smaller populations are more likely to have many individuals related to one another [47,48]. This is even more likely in species with aggregated pollen, as in *Echites*, so that one successful pollinator visit can produce a fruit full of seeds that are siblings, sharing the female parent, and are at least 50% related.

It is important in future work to consider the other ways that *Echites umbellatus* might be pollen limited [49]. Another reason for low fruit set may be a paucity of appropriate visitors, as was found for hawkmoth-pollinated flowers in the Atlantic Rainforest of Brazil [50]. As our own evening and night-time watches for flower visitors to *Echites umbellatus* in south Florida and the Bahamas revealed not a single visit, this is certainly possible. Measurements of pollen deposition on flowers in the field, as well as further observations of open flowers at all times of night, are required to determine how important this lack of visitation may be and if fruit set is limited due to lack of visitation or the deposition of incompatible pollen. In addition to pollen limitation, there may be resource limitation in the nutrient-poor rocklands of south Florida. Future experimental work may also take this factor into consideration.

## 5. Conclusions

Considering all populations together, we conclude that *Echites umbellatus* is mostly self-incompatible, though most populations have some individuals that can produce fruit via self-pollination. Fruits resulting from self-pollinations have fewer viable seeds than do fruits from pollinations with relatives, which in turn have fewer viable seeds than fruits from pollinations with unrelated individuals.

Even with ample visitation and pollination, flowers of a self-incompatible species may not receive the right kind of pollen required for fertilization and fruit production. The likelihood of receiving pollen from a related individual is higher in small populations existing in fragmented habitats, and that pollen is less likely to successfully fertilize the ovules in a flower. When *Echites umbellatus* occurs in fire-successional pine rockland habitats, post-fire pulses of nutrient input may promote growth and enhance flowering, as well as provide additional resources for fruit set. How such environmental fluctuations affect pollination and reproduction of this species will be interesting to explore.

Furthermore, plants persisting in natural area remnants are also less likely to be visited by their specialized pollinators, as insect numbers decline in proximity to urbanization. Knowledge of the breeding system of a native plant is important for understanding how it can successfully reproduce and persist. Studies of the kind described here, when coupled with field observation and experimentation, can provide information necessary for the conservation and continued existence of these plants in a changing world.

## Data Availability

Upon publication, data may be freely accessed from the FIU dataverse at https://doi.org/10.34703/gzx1-9v95/IFZWQ2 (accessed on 4 April 2024).
